# Therapeutic Response to Corticosteroids Remains a Valid Approach to Initial Management of Children With Idiopathic Nephrotic Syndrome

**DOI:** 10.3389/fped.2020.00533

**Published:** 2020-09-02

**Authors:** Deepti Narla, Agnieszka Swiatecka-Urban

**Affiliations:** Department of Nephrology, Children's Hospital of Pittsburgh of UMPC, University of Pittsburgh School of Medicine, Pittsburgh, PA, United States

**Keywords:** FSGS, minimal change disease, corticosteroids, steroid-sensitive, steroid-resistant, relapse, retrospective review, nephrotic syndrome (NS)

## Abstract

Complete remission of idiopathic nephrotic syndrome (INS) in response to corticosteroids has been widely adopted as an indicator of satisfactory long-term outcomes in pediatric patients. The approach was based on the results of studies conducted in the 1960s and 1970s. The studies found that corticosteroid-responsive minimal change disease (MCD) was the most frequent diagnosis in INS patients. In more recent years, studies have reported increased frequency of focal segmental glomerulosclerosis (FSGS) and primary corticosteroid resistance without a corresponding increase of FSGS. It became unclear whether withholding kidney biopsy before treatment with corticosteroids is still the best management practice. We performed a retrospective chart review at the UPMC Children's Hospital of Pittsburgh and identified patients who were referred for evaluation of edema or proteinuria between 2002 and 2014. We identified 114 pediatric patients with INS who were treated initially with a corticosteroid (prednisone or prednisolone) 2 mg/kg (max 60 mg)/day for 4–6 weeks followed by 2 mg/kg (max 60 mg) every other day for 4–6 weeks and had not received a corticosteroid-sparing agent before completing at least 8 weeks of the initial therapy. Corticosteroid resistance in pediatric INS patients was independently associated with the black race, older age at presentation (>8 years), and female sex. The majority of blacks who were resistant to corticosteroids had a tissue diagnosis of MCD. Among the whites who were steroid-resistant, MCD and FSGS were diagnosed in similar proportions of cases. Thus, the tissue diagnosis in could not predict the response to corticosteroids. Nineteen percent of whites with FSGS were steroid-sensitive and none of the blacks with FSGS responded to corticosteroids. These data suggest that the histologic diagnosis of FSGS could not rule out response to corticosteroids, at least, in the white patient population. In summary, our data demonstrate that at this time, the therapeutic response to corticosteroids continues to be a valid approach for the initial evaluation and therapy of children diagnosed with INS at our center. Future studies should evaluate the mechanisms of changing characteristics of pediatric INS. The specific role of patient demographics, ethnicity, as well as genetic and environmental factors could be evaluated by a prospective, multicenter study.

## Introduction

Idiopathic nephrotic syndrome (INS) is the most common form of nephrotic syndrome (NS) in children, representing more than 90% of cases between one and 10 years and 50% after 10 years of age (1). It is a non-inflammatory kidney disease resulting from alterations of the integrity of the glomerular capillary wall, leading to a triad of heavy proteinuria, hypoalbuminemia, and edema. The incidence worldwide varies widely between 1.2 and 16.9 cases per 100,000 children with the highest incidence observed on the Indian subcontinent compared to 2–3 cases per 100,000 children in most other regions (2). Males appear to be more affected than females at a ratio of 2:1 at a younger age, but this predominance fails to persist in adolescence.

The therapeutic approach to childhood INS is based on a series of studies that began with a collaborative effort sponsored by the International Study of Kidney Disease in Children (ISKDC) and Arbeitsgemeinschaft Fur Padiatrische Nephrologie (APN) in the 1960s and 1970s (3–7). The studies concluded that the therapeutic response to corticosteroids is useful for initial evaluation and therapy because histologic correlation showed that most INS patients had minimal change disease (MCD), usually sensitive to corticosteroids while <10% of patients had Focal Segmental Glomerulosclerosis (FSGS), usually resistant to corticosteroids (1, 8, 9). Hence, the response to corticosteroids has been widely adopted for the initial management and prevented unnecessary kidney biopsy in the majority of INS cases. However, many single-center studies conducted since the 1980s have reported that the frequency of FSGS increased by up to 47% in children of different ethnic backgrounds (10–15). Besides, the increased incidence of primary corticosteroid resistance without the corresponding increase of FSGS diagnosis was reported in the white population (16). Newer data demonstrate that relapses continue into adulthood in up to 42% of patients diagnosed with INS during childhood and correlate in those cases with the higher frequency of relapses during childhood and greater need for additional immunosuppressive medications (2, 17, 18). In this setting, it became unclear whether withholding the kidney biopsy, unless the patient fails to respond to corticosteroids, is still the best initial management practice to provide maximal therapeutic and diagnostic benefit at a minimal side effects' cost. Thus, our objective was to determine whether corticosteroids remain a valid initial approach in children with INS at our center.

## Methods

### Patients

We performed a retrospective chart review under an approved Institutional Review Board protocol PRO15020250 at the University of Pittsburgh and identified patients who were referred to the UPMC Children's Hospital of Pittsburg for evaluation of edema or proteinuria between 2002 and 2014. The hospital, located in Western Pennsylvania, is a tertiary care facility and a referral center for the entire Western Pennsylvania region. We excluded patients with familial FSGS, glomerulonephritis, and other secondary causes of NS. Patients between the age of 12 months and 18 years with INS who were treated initially with corticosteroids, either prednisone or prednisolone, 2 mg/kg (max 60 mg)/day for 4–6 weeks followed by 2mg/kg (max 60 mg) every other day for 4–6 weeks and had not received a corticosteroid-sparing agent before completing at least 8 weeks of the initial therapy were included in the study.

### Definitions

INS was defined according to the ISKDC criteria as heavy proteinuria (>40 mg/m^2^/h or >3.5 g/24 h in adolescents in a 24 h urine collection; or urine protein-to-creatinine ratio >2 mg/mg in a random urine sample), hypoalbuminemia (serum albumin <2.5 g/dL), and edema. Corticosteroid (steroid) responsive (SS) INS was classified according to the ISKDC, defined by the resolution of proteinuria within 8 weeks of corticosteroid therapy. The subsequent disease course defined the steroid-dependent (SD) patients who relapsed during therapy or within 14 days of therapy completion or frequently relapsing (FR) group that had two or more relapses within 6 months of the initial response or four or more relapses in any 12 months (19). Since the SD and FR patients were initial responders, they were included in the SS group. The steroid resistance (SR) was defined as a failure to achieve complete remission within 8 weeks of therapy.

### Pathology

Kidney biopsy was performed in patients who: (i) presented with NS at an age older than 10 years; (ii) had a significant elevation of serum creatinine at presentation that did not improve despite correction of intravascular volume; (iii) were SR; or (iv) those SS patients who became SD or FR. Renal tissue was examined by light, immunofluorescence, and electron microscopy (LM, IFM, and EM, respectively) and was interpreted by a pediatric pathologist. Minimal change disease (MCD) was defined by the presence of foot process effacement on EM and the absence of any conspicuous lesions on LM. Specimen positive for isolated C1q or IgM staining on IFM in patients with normal C3 and C4 complement levels was considered a variant of MCD (20, 21). Specimen with mesangial hypercellularity (MH) was considered a separate category of INS. Focal segmental glomerulosclerosis (FSGS) was defined as the presence of segmental sclerosis in at least one glomerulus with or without tubular atrophy or interstitial fibrosis. Membranous nephropathy (MN) was characterized by homogeneous thickening of the glomerular capillary walls on periodic acid-Schiff staining and increased basement membrane deposition with characteristic “spike and hole” configuration on methenamine silver staining on LM, confirmed by demonstration of electron-dense deposits in the glomerular basement membrane and subepithelial regions by EM (22).

### Statistical Analysis

Data from groups of patients are reported as mean ± SD or as percentages (frequency of observation in a particular group). Statistical analysis was performed by the Student *t*-test or Fisher exact test. Multiple logistic regression was performed to explain the relationship between steroid response, age at presentation, race, and sex in a multivariable model. Statistical significance was defined by a two-tailed *p* < 0.05.

## Results

We identified 153 patients who were diagnosed with NS between 2002 and 2014. Of these patients, 114 met the inclusion criteria and were included in the study. The remaining patients were excluded for the following reasons: familial FSGS (*N* = 19), incomplete records (*N* = 9), treatment with a corticosteroid-sparing agent within 8 weeks of the initial presentation (*N* = 8), or lack of treatment (*N* = 3). All patients included in the study were treated initially with a corticosteroid (prednisone or prednisolone) as a single agent at a dose of 2 mg/kg (max 60 mg)/day for 4–6 weeks followed by 2 mg/kg (max 60 mg) every other day for 4–6 weeks. All except four patients were referred before initiating corticosteroid therapy. The mean age of our entire population was 6.7 ± 4.1 years with a range of 12 months to 16.9 years. The study population consisted of 48% females and 52% males. The major ethnic groups were whites (*N* = 90; 79%) and blacks (*N* = 19; 17%). Two biracial patients were included in the black cohort. Three patients were Asians and one was Hispanic. The ethnic background of one patient was not disclosed. The demographic characteristics of our study group is compared to the ISKDC population in Table 1.

**Table 1:** Demographic data of the study cohort and comparison to the ISKDC population (1, 5, 8).

**Category**	**Our population**	**ISKDC population**
Number of patients	114	521
Age (mean ± SD) range	6.7 ± 4.1 years 12 months – 16.9 years	4.8 ± 2.9 10 months – 14.8 years
Females	55 (48%)	39%
Males	59 (52%)	61%
Whites	79% (*N* = 90)	Not available
Blacks	17% (*N* = 19)	Not available
Hispanics	1% (*N* = 1)	Not available
Asians	2% (*N* = 3)	Not available
Not Disclosed	1% (*N* = 1)	Not available
North America	100%	35.7%
Europe	0%	47.8%
Japan and Hong Kong	0%	16.5%

Table 2 shows the distribution of the initial responses to steroids in our study population. SR INS was observed in 36% of patients in our study, compared to 12% reported by the ISKDC (3, 8). Between 2002 and 2008, 24 (42%) patients were diagnosed with SR INS and between 2009 and 2014, 18 (32%) of patients were diagnosed with SR INS. There was no statistically significant difference in the proportion of patients diagnosed with SR INS during 2002–2007 and 2008–2014 (Fisher exact, *p* = 0.33). SR INS was observed in 27 (30%) of whites compared to 14 (74%) in blacks. In a univariate model, SR INS was associated with the black race (Fisher exact, *p* = 0.0006). As shown in Table 2, the SR was twice as common in patients who presented with INS at age 8 years or above, compared to those diagnosed at a younger age (60 vs. 29%; *p* = 0.004). Females presented at an older age than males (7.2 ± 4.2 vs. 5.2 ± 4.0; *p* = 0.01) and the SR INS was almost twice as common in females, compared to males (49 vs. 25%; *p* = 0.01; Table 2). In a multivariable model, the black race, age 8 years or above at presentation, and female sex were independently associated with the SR INS (Table 3).

**Table 2:** Distribution of responses to corticosteroids (steroids) by race, age, and gender.

	**Response to** **steroids**	***N* (%)** ***N = 110***
Total	SS	73 (64%)
	SR	41 (36%)
Whites	SS	63 (70%)
	SR	27 (30%)
Blacks	SS	5 (26%)
	SR	14 (74%)
Age <8	SS	60 (71%)
	SR	24 (29%)
Age >8	SS	12 (40%)
	SR	18 (60%)
Males	SS	44 (75%)
	SR	15 (25%)
Females	SS	28 (51%)
	SR	27 (49%)

*Steroid resistance (SR) was specifically associated with the black race (fisher exact, p-value = 0.0006), the age of 8 years or above at presentation (Fisher exact, p = 0.004), and female sex (Fisher exact, p = 0.01). SS, steroid-sensitive*.

**Table 3:** Multiple logistic regression for age at initial presentation, sex, and responses to steroids.

**Association of** **SR with:**	**Odds ratio**	**Standard error**	***P*-value**	**95% confidence** **interval**
Age >8years	2.79	0.49	0.03	1.08–7.37
Female sex	3.91	0.48	0.004	1.78–10.50
Black race	8.14	0.63	0.0009	2.52–30.84

*Black race, age 8 years or above at presentation, and female sex were independently associated with steroid resistance (SR)*.

A biopsy was performed in 85 out of 114 patients because of one or more of the following characteristics: (i) presentation at an age older than 10 years; (ii) significant elevation of serum creatinine at initial presentation that did not improve after correction of intravascular volume; (iii) SR course; or (iv) SD or FR course after the initial SS presentation. Table 4 shows the distribution of histologic characteristics in our patients. MCD and FSGS were the most common histologic diagnoses, present in 73 and 24% of all biopsy specimens, respectively. According to the ISKDC studies, MCD was the most likely histologic diagnosis in SS patients presenting at a younger age who did not become SD or FR. Assuming that all children with SS INS who were not biopsied had MCD (presumptive MCD), the total incidence of MCD in our study was 80%, comparing to 76–88% previously reported by the ISKDC (1, 5). In our study, the incidence of FSGS was twice as high, compared to the ISKDC (18 vs. 7–8%) (1, 5). Moreover, the SS INS characterized 76% of our patients with MCD (biopsy-confirmed and presumptive) and 20% of patients with FSGS, compared to 93–98% and 17–30%, respectively in the ISKDC studies (3, 4). The proportion of SR patients diagnosed with FSGS in our study was identical to the ISKDC (39%).

**Table 4:** Distribution of the histopathological lesions according to response to steroids and race.

**Histopathology**	**MCD**	**FSGS**	**MN**
All patients *N* (%) (*N* = 85)	62 (73%)	20 (24%)	3 (3%)
SS (*N* = 44)	40 (91%)	4 (9%)	0 (0%)
SR (*N* = 41)	22 (54%)	16 (39%)	3 (7%)
Whites	46 (71%)	16 (24 %)	3 (5%)
Blacks	14 (82%)	3 (18%)	0 (0%)

*SS, steroid-sensitive; SR, steroid-resistant; MCD, minimal change nephrotic syndrome; FSGS, focal segmental glomerulosclerosis; MN, membranous nephropathy*.

As shown in Table 4, similar proportions of black and white patients in our study were diagnosed with FSGS (18 and 24%, respectively). We had limited representation from other ethnicity, with one Hispanic diagnosed with MCD and two Asians with MCD and FSGS, each. FSGS is generally considered SR so it came as a surprise that only 18% or blacks were diagnosed with FSGS although 74% were SR (Table 2 vs. Table 1). Among the black SR patients (*N* = 13), FSGS was diagnosed in 23% and MCD was diagnosed in the remaining 77% of patients (Table 4). Among the white SR patients (*N* = 27), MCD and FSGS were found in 44% each and MN in 12% of patients (Table 4). Of the 20 cases of FSGS in our study, 16 were SR (80%) and 4 (20%) were SS. None of the black FSGS patients (*N* = 3) were SS while 19% of white FSGS patients (*N* = 3/16) were SS. MN was diagnosed only in SR whites. Taken together these data show that MCD was characterized by more steroid resistance in our patients, compared to the ISKDC population. The tissue diagnosis of MCD could not predict the response to corticosteroids in the black and white patients in our study. Moreover, the diagnosis of FSGS could not rule out response to corticosteroids, at least, in the white patient population in our study.

## Discussion

Our retrospective review shows an independent association of pediatric SR INS with the black ethnicity, older age at presentation (>8 years), and female sex. Despite the similar incidence of MCD compared to the ISKDC group, our cohort was characterized by a higher incidence of SR among the black and white patients with MCD. Since the majority of black SR patients had MCD, the histologic diagnosis could not predict the disease course ahead of corticosteroid therapy in this patient population. The distribution of MCD and FSGS was similar among the white SR patients, indicating that the tissue diagnosis of MCD could not predict the response to corticosteroids in this patient population. Since 19% of the white FSGS patients were SS, the tissue diagnosis of FSGS before the initiation of therapy could not predict the response to corticosteroids in this group. Our results are similar to those reported form South Africa by Bhimma et al. who noted that 56% (*N* = 18/32) of black children with biopsy-confirmed MCD were SR, consistent with the lack of correlation between the tissue diagnosis and response to steroids (23). In the same study, all black patients with FSGS (*N* = 67) were SR. All black FSGS patients in our study (*N* = 3) were also SR. Taken together, in spite of higher proportion of SR among the MCD patients in our study, compared to the ISKDC, our findings reaffirm the ISKDC results and demonstrate that, at present, the therapeutic response to corticosteroids is a valid approach for the initial evaluation and therapy for INS in children with the black and white ethnic background.

There were differences in the temporal, demographic, and ethnic characteristics and the male-to-female ratio between the patients reported by us and ISKDC. Our retrospective study performed three decades after the ISKDC, represents primarily white and black patients from a single US center while the ISKDC cohort comprised a multiethnic group from 12 countries in Europe, Asia, and North America (3, 8). Asian patients were underrepresented in our cohort, compared to the ISKDC group (2 vs. 16.5%). The ISKDC studies did not report separate results for the major ethnic groups. Hence, it remains unclear whether the ethnic background influenced the results reported by ISKDC the way it did in our study. When considering the entire study populations, the incidence of SR in our cohort fared worse when compared to the ISKDC group (36 vs. 12–13%). The difference could be attributed to a higher incidence of FSGS (18 vs. 7–8%) and a higher proportion of SR among patients with MCD (24 vs. <10%) in our study, compared to ISKDC. The male-to-female ratio was lower in our study, compared to ISKDC (1.1:1 and 1.6:1, respectively). The mean age at presentation was higher by almost 2 years in our patient population and the difference could contribute, at least in part, to the absence of male predominance in our study population since the predominance decreases among those patients who present with INS during later childhood and adolescence (Table 1). Although our female patients presented with INS later than males, the female sex was associated with SR independently of the age at presentation (Table 2 vs. Table 3). The association between female sex and SR has been reported by Mortazavi and Khiavi who studied 165 children from a single center in Tabriz, Iran (24). The study supports our conclusion that female sex is an independent risk of SR in some patient populations because females presented with INS at an age similar to males and almost identical to the ISKDC cohort. By contrast, a single-center study from Bosnia reported no association between sex and SR while studies from Saudi Arabia, France, and an Iranian center in Tehran reported that male sex was associated with both, SR and SS INS (13, 25–27). Cumulatively, the above studied performed in INS patients of different ethnic backgrounds on different continents indicate that the association between sex and SR is poorly understood.

Several centers reported an increasing incidence of SR and FSGS in children during the years following ISKDC studies. SR INS was observed in 36% of our entire cohort and in 31% of white patients reported by Banaszak and Banaszak compared to 22% of the ISKDC cohort reported by Abramowicz (3) and Banaszak and Banaszak (16). Compared to ISKDC that reported FSGS in 7% of the entire cohort, it was diagnosed in 22% (*N* = 33/152) of patients in a study by Bonilla-Felix et al. between 1978 and 1997, 18% (*N* = 29/159) of patients reported by Filler *et* al. between 1985 and 2002, 36% patients reported by Banaszak and Banaszak between 1996 and 2005, and 18% of patients in our study (10, 16, 28). Interestingly, the incidence of FSGS in the study by Bonilla-Felix and Filler increased significantly over the study period (23% before 1990 to 47% after 1990 and from 11% during the first half of the study to 25% during the second half of the study) (10, 28). By contrast, the incidence of SR and FSGS trended down during the second half of our study (42 vs. 32%).

Similar proportions of black and white patients in our study were diagnosed with FSGS (18 and 24%, respectively). These results differ from those by Bonilla-Felix where the incidence of FSGS was significantly higher in blacks, compared to whites (47 and 18%, respectively) (10). The different incidence of FSGS between our studies could be reconciled, at least in part, by the exclusion of 19 black patients with a familial form of FSGS in our study. If the patients with familial FSGS were included, the incidence of FSGS in the black cohort would increase to 54% in our study. The increased incidence of FSGS was previously reported in a group combining the black and Hispanic children (29). However, it is impossible to compare the group with our cohort of exclusively black patients.

We acknowledge that the design, a retrospective review from a single center, is a weakness of our study and our conclusions may need to be validated in prospective studies and may vary in different ethnic backgrounds. There are several strengths of our study. First, it reconciles the multicenter and multiethnic ISKDC data collected three decades earlier with the recent data from white and black patients in our region. This is important because the ISKDC studies did not report separate results for the major ethnic groups. For example, we show an independent association of SR INS with the black ethnicity, older age at presentation, and female sex. Second, similar to other post-ISKDC single center studies, we report changing characteristics of INS with higher incidence of SR and FSGS that, unlike the older reports, has plateaued over our study period. Third, we show that despite the changing characteristics, the therapeutic response to corticosteroids is a valid approach for the initial evaluation and therapy for INS in the black and white children. We would like to emphasize a different reason why the therapeutic response to corticosteroids is useful for the initial evaluation and therapy for childhood INS in our study and the ISKDC studies. They concluded that it was because histologic correlation showed that most INS patients had MCD, usually sensitive to corticosteroids while only small proportion of patients had FSGS, usually resistant to corticosteroids. By contrast, our data indicate that it is because the histologic diagnosis of either MCD or FSGS in black and white children with INS does not predict the response to corticosteroids. Thus, our single center retrospective review provides important updates on the childhood INS characteristic and emphasizes the continued need to study the condition in different ethnic backgrounds and over time.

Mechanisms of the changing characteristics of INS mechanism remain elusive. We have recently learned how air pollution increases the risk of chronic kidney disease in carriers of the *APOL1* high-risk genotype (30). It remains to be determined whether an interplay between genetic and environmental risk factors contributes to the evolving characteristics of INS. Until the mechanisms are elucidated and preventive strategies validated, we propose that the therapeutic response to corticosteroids continues to be an appropriate approach for the initial evaluation and therapy for children with INS at our center.

## Data Availability Statement

The raw data supporting the conclusions of this article will be made available by the authors, without undue reservation.

## Ethics Statement

The studies involving human participants were reviewed and approved by the University of Pittsburgh IRB protocol PRO15020250. Written informed consent from the participants' legal guardian/next of kin was not required to participate in this study in accordance with the national legislation and the institutional requirements.

## Author Contributions

DN and AS-U designed the study, analyzed the data, and wrote the manuscript. The authors read and approved the final manuscript. All authors contributed to the article and approved the submitted version.

## Conflict of Interest

The authors declare that the research was conducted in the absence of any commercial or financial relationships that could be construed as a potential conflict of interest.

## References

[1] International Study of Kidney Disease in Children. Nephrotic syndrome in children: Prediction of histopathology from clinical and laboratory characteristics at time of diagnosis. Kidney Int. (1978) 13:159–65. 10.1038/ki.1978.23 713276

[2] Noone DG Iijima K Parekh R. Idiopathic nephrotic syndrome in children. Lancet. (2018) 392:61–74. 10.1016/S0140-6736(18)30536-1 29910038

[3] Abramowicz M Barnett HL Edelmann C. M. Jr Greifer I Kobayashi O Controlled trial of azathioprine in children with nephrotic syndrome. A report for the international study of kidney disease in children. Lancet. (1970) 1:959–61. 10.1016/S0140-6736(70)91093-7 4191931

[4] Churg J Habib R White RH Pathology of the nephrotic syndrome in children: a report for the International Study of Kidney Disease in Children. Lancet. (1970) 760:1299–302. 10.1016/S0140-6736(70)91905-7 4193942

[5] White RH Glasgow EF Mills RJ. Clinicopathological study of nephrotic syndrome in childhood. Lancet. (1970) 1:1353–9. 10.1016/S0140-6736(70)91268-7 4194123

[6] Rothenberg MB Heymann W. The incidence of the nephrotic syndrome in children. Pediatrics. (1957) 19:446–52. 13408023

[7] The primary nephrotic syndrome in children. Identification of patients with minimal change nephrotic syndrome from initial response to prednisone. A report of the International Study of Kidney Disease in Children. J Pediatr. (1981) 98:561–4. 10.1016/S0022-3476(81)80760-3 7205481

[8] Tarshish P Tobin JN Bernstein J Edelmann CM Jr. Prognostic significance of the early course of minimal change nephrotic syndrome: report of the International Study of Kidney Disease in Children. J Am Soc Nephrol. (1997) 8:769–76. 9176846 10.1681/ASN.V85769

[9] Schulman SL Kaiser BA Polinsky MS Srinivasan R Baluarte HJ. Predicting the response to cytotoxic therapy for childhood nephrotic syndrome: superiority of response to corticosteroid therapy over histopathologic patterns. J Pediatr. (1988) 113:996–1001. 10.1016/S0022-3476(88)80570-5 3193322

[10] Bonilla-Felix M Parra C Dajani T Ferris M Swinford RD Portman RJ. Changing patterns in the histopathology of idiopathic nephrotic syndrome in children. Kidney Int. (1999) 55:1885–90. 10.1046/j.1523-1755.1999.00408.x 10231451

[11] Kim JS Bellew CA Silverstein DM Aviles DH Boineau FG Vehaskari VM. High incidence of initial and late steroid resistance in childhood nephrotic syndrome. Kidney Int. (2005) 68:1275–81. 10.1111/j.1523-1755.2005.00524.x 16105061

[12] Chesney R. The changing face of childhood nephrotic syndrome. Kidney Int. (2004) 66:1294–302. 10.1111/j.1523-1755.2004.00885.x 15327442

[13] Kari JA. Changing trends of histopathology in childhood nephrotic syndrome in western Saudi Arabia. Saudi Med J. (2002) 23:317–21. 11938425

[14] Gulati S Sharma AP Sharma RK Gupta A. Changing trends of histopathology in childhood nephrotic syndrome. Am J Kidney Dis. (1999) 34:646–50. 10.1016/S0272-6386(99)70388-4 10516344

[15] Srivastava T Simon SD Alon US. High incidence of focal segmental glomerulosclerosis in nephrotic syndrome of childhood. Pediatr Nephrol. (1999) 13:13–8. 10.1007/s004670050555 10100283

[16] Banaszak B Banaszak P. The increasing incidence of initial steroid resistance in childhood nephrotic syndrome. Pediatr Nephrol. (2012) 27:927–32. 10.1007/s00467-011-2083-7 22231438 PMC3337414

[17] Fakhouri F Bocquet N Taupin P Presne C Gagnadoux MF Landais P. Steroid-sensitive nephrotic syndrome: from childhood to adulthood. Am J Kidney Dis. (2003) 41:550–7. 10.1053/ajkd.2003.50116 12612977

[18] Kyrieleis HA Lowik MM Pronk I Cruysberg HR Kremer JA Oyen WJ. Long-term outcome of biopsy-proven, frequently relapsing minimal-change nephrotic syndrome in children. Clin J Am Soc Nephrol. (2009) 4:1593–600. 10.2215/CJN.05691108 19808243 PMC2758253

[19] Group KDIGOK GW KDIGO clinical practice guideline for glomerulonephritis. Kidney Int. (2012) 2:139–274. 10.1038/kisup.2012.12

[20] Markowitz GS Schwimmer JA Stokes MB Nasr S Seigle RL Valeri AM. C1q nephropathy: a variant of focal segmental glomerulosclerosis. Kidney Int. (2003) 64:1232–40. 10.1046/j.1523-1755.2003.00218.x 12969141

[21] Zeis PM Kavazarakis E Nakopoulou L Moustaki M Messaritaki A Zeis MP. Glomerulopathy with mesangial IgM deposits: long-term follow up of 64 children. Pediatr Int. (2001) 43:287–92. 10.1046/j.1442-200x.2001.01396.x 11380926

[22] Keri KC Blumenthal S Kulkarni V Beck L Chongkrairatanakul T. Primary membranous nephropathy: comprehensive review and historical perspective. Postgrad Med J. (2019) 95:23–31. 10.1136/postgradmedj-2018-135729 30683678

[23] Bhimma R Coovadia HM Adhikari M. Nephrotic syndrome in South African children: changing perspectives over 20 years. Pediatr Nephrol. (1997) 11:429–34. 10.1007/s004670050310 9260239

[24] Mortazavi F Khiavi YS. Steroid response pattern and outcome of pediatric idiopathic nephrotic syndrome: a single-center experience in northwest Iran. Ther Clin Risk Manag. (2011) 7:167–71. 10.2147/TCRM.S19751 21691587 PMC3116804

[25] Otukesh H Otukesh S Mojtahedzadeh M Hoseini R Fereshtehnejad SM Riahi Fard A. Management and outcome of steroid-resistant nephrotic syndrome in children. Iran J Kidney Dis. (2009) 3:210–7. 19841524

[26] Mekahli D Liutkus A Ranchin B Yu A Bessenay L Girardin E. Long-term outcome of idiopathic steroid-resistant nephrotic syndrome: a multicenter study. Pediatr Nephrol. (2009) 24:1525–32. 10.1007/s00467-009-1138-5 19280229

[27] Pokrajac D Kamber AH Karasalihovic Z. Children with steroid-resistant nephrotic syndrome: a single -center experience. Mater Sociomed. (2018) 30:84–8. 10.5455/msm.2018.30.84-88 30061794 PMC6029918

[28] Filler G Young E Geier P Carpenter B Drukker A Feber J. Is there really an increase in non-minimal change nephrotic syndrome in children? Am J Kidney Dis. (2003) 42:1107–13. 10.1053/j.ajkd.2003.08.010 14655180

[29] Ingulli E Tejani A. Racial differences in the incidence and renal outcome of idiopathic focal segmental glomerulosclerosis in children. Pediatr Nephrol. (1991) 5:393–7. 10.1007/BF01453661 1911111

[30] Paranjpe I Chaudhary K Paranjpe M O'Hagan R Manna S Jaladanki S. Association of APOL1 risk genotype and air pollution for kidney disease. Clin J Am Soc Nephrol. (2020) 15:401–3. 10.2215/CJN.11921019 32079610 PMC7057301

